# Levodopa does not affect expression of reinforcement learning in older adults

**DOI:** 10.1038/s41598-019-42904-5

**Published:** 2019-04-23

**Authors:** J. P. Grogan, H. K. Isotalus, A. Howat, N. Irigoras Izagirre, L. E. Knight, E. J. Coulthard

**Affiliations:** 1 University of Bristol, Bristol, UK; 2 University Hospitals Bristol, Bristol, UK; 3 North Bristol NHS Trust, Bristol, UK

**Keywords:** Long-term memory, Operant learning

## Abstract

Dopamine has been implicated in learning from rewards and punishment, and in the expression of this learning. However, many studies do not fully separate retrieval and decision mechanisms from learning and consolidation. Here, we investigated the effects of levodopa (dopamine precursor) on choice performance (isolated from learning or consolidation). We gave 31 healthy older adults 150 mg of levodopa or placebo (double-blinded, randomised) 1 hour before testing them on stimuli they had learned the value of the previous day. We found that levodopa did not affect the overall accuracy of choices, nor the relative expression of positively or negatively reinforced values. This contradicts several studies and suggests that overall dopamine levels may not play a role in the choice performance for values learned through reinforcement learning in older adults.

## Introduction

Dopamine has been heavily implicated in reinforcement learning^1–3^, and recently evidence has shown that dopamine also affects later choices based on these learned values^4–6^. However, unpicking the relative contribution of dopaminergic neurons during encoding, consolidation and retrieval stages of memory is often confounded by relatively long duration of action of medications.

### Exogenous dopamine administration biases consolidation or retrieval in Parkinson’s disease

An early study showed that if Parkinson’s disease (PD) patients were given their dopaminergic medication before completing a reinforcement learning task they learned better from positive than negative feedback^1^. The opposite pattern was shown if they were withdrawn from their dopaminergic medication prior to learning. However, the differences were not apparent during the learning trials themselves. Instead, after learning, all the combinations of stimuli were presented without feedback to see whether participants had learned the relative value of the symbols via positive or negative reinforcement. It was only on this latter choice phase that the differences between medication states were seen, which raised the possibility that dopamine does not actually affect the learning process, but a separate process invoked when choosing stimuli based on their learned values. This could be a retrieval process for the learned values, or a decision process on the retrieved values.

When learning and choice trials were separated by a delay, which allowed PD patients to learn off medication and be tested on or off medication, medication state during learning had no effect on expression of positive or negative reinforcement, but dopaminergic state during the choices did^4^. This was accompanied by fMRI signals in the ventro-medial prefrontal cortex and nucleus accumbens tracking the value of stimuli only when PD patients were on medication. This suggested that dopamine improved the retrieval and comparison of the learned values.

Similarly, when PD patients learned a set of stimulus-stimulus associations, and only had the rewards mapped onto these stimuli after they had finished learning, they still showed a bias towards the most rewarded stimuli if they were on their medications during the entire session^5^. This demonstrated that the reward bias could be induced even when reward learning did not take place. Thus, dopamine appeared to affect value-based decision making, with a bias towards rewarding outcomes.

However, other studies have failed to find effects of dopamine during choice performance, with dopamine during testing 24 hours after reinforcement learning not affecting the change in accuracy from the learning trials^7,8^. One of these studies^7^ also found that PD patients on their dopaminergic medications during learning had poorer learning than those off medication. However, this task was a deterministic feedback task, rather than a probabilistic feedback task as used in most other studies, which may have different learning mechanisms due to the lack of stochasticity.

### Effect of dopamine administration in healthy young adults

While patients with Parkinson’s are known to be dopamine-depleted without medication, healthy young adults are usually considered to have optimal levels of dopaminergic activity for brain processing. Given the dopamine overdose hypothesis^9^ posits an optimal level of dopaminergic function, where both increases or decreases to this level impair functioning, one would predict distinct effects of dopamine administration on healthy young people compared to older people with relative dopaminergic loss^10^ and people with Parkinson’s disease who have more profound dopaminergic loss. Using the deterministic stimulus-response task mentioned above, healthy young participants were worse at learning after 100 mg levodopa^11^. Likewise, pramipexole, a D2 agonist, impaired learning on the same task^12^. This could be explained by the increased dopaminergic activity tipping people over the peak of the inverted U-shaped response posited by the dopamine overdose hypothesis^13^.

A dopamine D2/3 receptor antagonist given to young adults during a probabilistic reward/punishment task did not affect the earlier stages of learning, but impaired performance at the later stages of the learning task, though only for the rewarded stimuli^6^. Computational modelling demonstrated an effect of dopamine on the choice parameter for the reward stimuli, but not for the punishment stimuli, or the learning rates, suggesting that the effect was not driven by learning from the feedback. This points to a D2/3 contribution to consolidation or retrieval of rewarded information in healthy young adults.

### Effects of exogenous dopamine in older adults

When healthy older participants were given levodopa before a reward/punishment learning task, they showed better performance on the reward trials, but no difference on the punishment trials, when compared against a haloperidol (D2 inverse agonist) group^14^. Neuroimaging revealed that levodopa increased the striatal reward prediction errors for reward trials but did not affect aversive prediction errors from the punishment trials. If contrasted with Eisenegger *et al*.^6^, it suggests that dopamine contributes to the reward prediction errors during learning, and that D2 receptors are important for the selection of actions, but not the learning from them. However, these studies used tasks with only learning trials, and used analysis techniques to try to separate out the influence of the drug on learning and choice selection within that. While other studies with positive and negative outcomes have used post-learning phases to remove the influence of feedback affecting choices^15–17^, these have not been used with dopaminergic manipulations to our knowledge.

Here, we used a separate choice phase on a reinforcement learning task which had no feedback, and thus tested choice selection only, to assess how levodopa affects the expression/retrieval of positive and negative learning. We chose levodopa as the drug as it is the most commonly prescribed dopaminergic treatment in PD patients, and has previously shown effects on a similar task^14^ in healthy adults. In order to isolate the effects of dopamine administration on choice performance from learning or consolidation, we gave this choice phase 24 hours after initial learning and gave participants either 150 mg levodopa or a placebo 1 hour before.

## Methods

### Participants

Thirty-five healthy older adults were recruited from Join Dementia Research and the ReMemBr Group Healthy Volunteer database. One participant was excluded due to glaucoma (contraindication), and three withdrew before completing both conditions. Thirty-one participants completed both conditions.

Participants were native English speakers over 65 years old with normal or corrected vision. They had no neurological or psychiatric disorders and did not have any of the contraindications for the study drugs Domperidone and Madopar (levodopa; see Supplementary Materials 1). They were not taking any monoaminergic medications, or any drugs listed in the Summary of Product Characteristics for Domperidone or Madopar. Demographic details are provided in Table 1.

**Table 1:** Demographics and questionnaires statistics.

Measure	Mean	SD	Range
N (Male: Female)	31 (14:17)		
Age	71.23	7.41	65–92
Years of Education	14.42	3.45	10–24
MoCA	26.19	3.10	18–30
DASS Total	11.29	10.12	1–39
DASS-D	3.84	4.51	0–18
DASS-A	2.10	2.47	0–11
DASS-S	5.35	4.10	0–14
BIS	57.53	9.01	38–73
LARS	−26.65	5.45	−34–14

The means, standard deviations (SD) and ranges of the demographic and questionnaire data for the participants. Montreal Cognitive Assessment (MoCA) of less than 24 suggests cognitive impairment, Barratt Impulsivity Scale (BIS) of 72 or higher suggests high impulsivity, Lille Apathy Rating Scale (LARS) scores above −22 suggest apathy, and a Depression Anxiety Stress Scale (DASS) above 21, 15, and 26 suggest severe depression, anxiety and stress, respectively.

Participants were tested at Southmead Hospital, Bristol, UK. All participants gave written informed consent at the start of each testing session, in accordance with the Declaration of Helsinki. Ethical approval was granted by University of Bristol Faculty Research Ethics Committee. All procedures were in accordance with Good Clinical Practice and HRA and ethical regulations.

### Design

A double-blinded, within-subjects, randomised placebo-controlled design was used. The two drugs were 10 mg suspension of Domperidone and 187.5 mg Madopar (37.5 mg benserazide + 150 mg levodopa) dispersible, both mixed with diluted squash, and the placebos were diluted squash, with a Vitamin C tablet dissolved in one to mimic the residue left by the Madopar dispersible tablet. The levodopa dose was chosen to match previous studies which have found effects of dopamine on reinforcement learning tasks^18,19^.

Domperidone is a peripheral dopamine D2 receptor antagonist, given 1 hour before levodopa to counter the nausea sometimes caused by it. The drugs and placebos were prepared by a lab member not otherwise involved in the study.

### Tasks

The reinforcement task was adapted from Pessiglione *et al*.^14^, and is referred to as the GainLoss task. It was run using Matlab r2015 and Psychtoolbox-3^20–22^ on Dell Latitude 3340 laptops. Links to download the code are provided in the Data Availability section in this manuscript.

**Figure 1:**
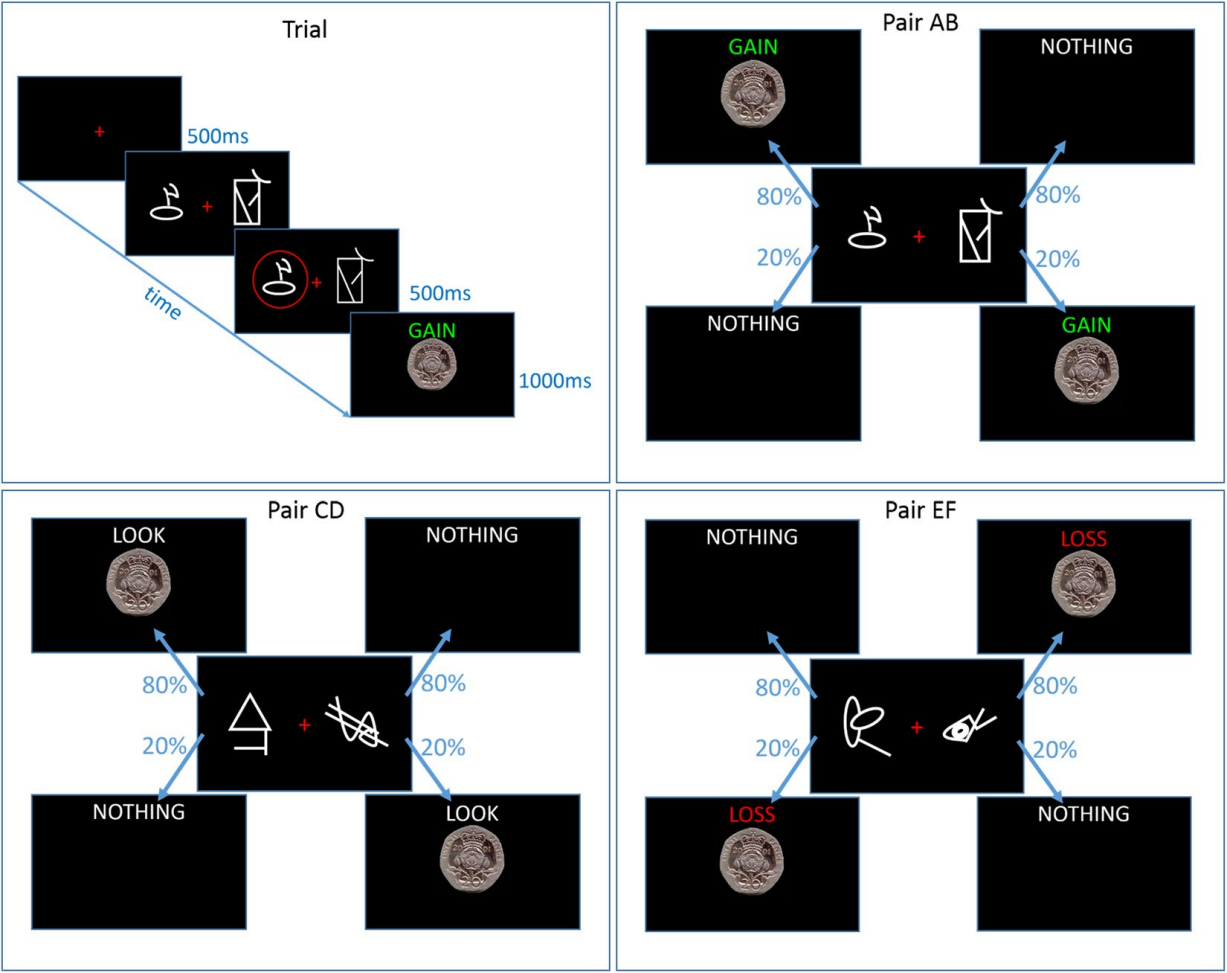
Diagram of the GainLoss experiment learning trials. Top left shows a sample Gain trial, and the other three panels show the outcome probabilities for the symbols in each pair (representative symbols shown here).

In this task, volunteers were instructed to attempt to win as much money as possible. During learning, on each trial one of three pairs of symbols (Fig. 1) was shown on the computer screen until the participants selected one symbol using the keyboard (there was no response deadline). After this their selection was circled in red for 500 ms. This was followed by one of four outcomes presented on the screen for 1000 ms: GAIN 20 pence; LOSE 20 pence; LOOK at a 20 pence piece; or NOTHING. The outcome was determined probabilistically, with symbol A in the Gain pair resulting in ‘GAIN’ on 80% of trials, and ‘NOTHING’ on 20%, and vice versa for symbol B in the Gain pair. In the Look pair, symbol C resulted in a ‘LOOK’ outcome 80% of the time, and ‘NOTHING’ 20% of the time (vice versa for symbol D), and in the Loss pair symbol F had an 80% chance of resulting in a ‘LOSS’ and 20% chance of ‘NOTHING’ (vice versa for symbol E). The outcome was displayed for 1000 ms, which was followed by a fixation cross for 500 ms before the onset of the next trial.

The learning was preceded by a practice block of 30 trials (10 for each pair, using different symbols to the learning blocks), followed by two blocks of 90 learning trials (30 trials per pair). Choice performance was measured by showing all symbols in all combinations six times (e.g. AB, AC, AD…, 15 pairs in total, 6 repetitions of each pairs, 90 trials in total) without the outcomes shown. The stimuli were presented for the same duration as in the learning trials, except without the outcome screen. Choice performance was assessed immediately after learning, after a 30-minute delay, and 24 hours later. Different sets of stimuli were used for each condition, the order of which was randomised across participants.

An episodic verbal learning task was also learned on day 1. Participants read aloud a list of 100 words and were tested 30 minutes and 24 hours later with the remember-know paradigm. Several questionnaires and paper tests were also given; digit span^23^ and the St. Mary’s Hospital Sleep Questionnaire^24^ (SMHSQ) were given each day, and the Montreal Cognitive Assessment^25^ (MoCA), Barratt Impulsivity Scale^26^ (BIS), Lille Apathy Rating Scale^27^ (LARS), Depression Anxiety Stress Scale^28^ (DASS) and Rational-Experiential Inventory^29^ (REI) were given once each on day 1 or day 3 (i.e. not after drug or placebo). The digit span measures were reported elsewhere^30^, but in brief levodopa did not affect working memory capacity but did impair accuracy on manipulation components.

### Procedure

**Figure 2:**
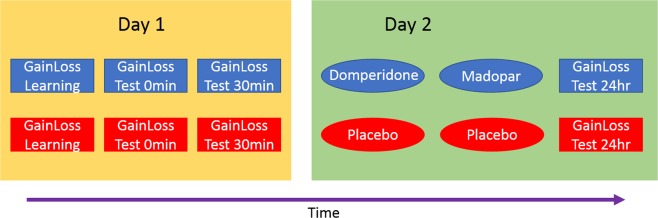
Timeline of experimental conditions. Each condition was identical except that in one pair of days participants received the drugs (blue) 1 hour before testing on Day 2, and on the other received the placebos (red) before testing. The order of drug and placebo condition was randomised across participants.

Participants completed four testing sessions, arranged into two pairs of days (see Fig. 2). On day 1, participants gave consent and were fully screened for all contraindications and interactions for the study drugs (Domperidone and Madopar), and Vitamin C, which was used in the placebo. They then learned the cognitive tasks and completed some of the questionnaires during the 30-minute delay before being tested on the tasks.

On day 2, participants again gave consent and continued eligibility was confirmed. Baseline blood pressure and heart rate was recorded before the Domperidone (or placebo; double-blinded) was administered. Thirty minutes later their blood pressure and heart rate were measured again, and the levodopa (or placebo) was given. Blood pressure and heart rate were also recorded 30 and 60 minutes later. One hour after the levodopa (or placebo) was administered, participants completed the GainLoss and remember-know tasks, digit span and SMHSQ. They then learned another list of words to test encoding effects of dopamine on long term memory, and memory was tested immediately, and over the phone 1, 3 and 5 days later.

Days 3 and 4 were identical to days 1 and 2, with the exception of the drug/placebo. On the last phone test after day 4, participants were asked which day they thought they received the drugs to assess blinding success.

### Data analysis

Selection of the symbol that was more likely to lead to the highest value of the two shown was considered the optimal response, regardless of the outcome actually given on that learning trial (e.g. if they select symbol A, the 80% Gain symbol, this is considered optimal even it results in ‘NOTHING’ on that particular trial). For the Look pair, symbol C (80% LOOK) was treated as optimal when it was against ‘NOTHING’ even though neither outcome had monetary value. The Look symbols were considered optimal against the Loss symbols, while the Gain symbols were considered optimal against the Look symbols.

**Figure 3:**
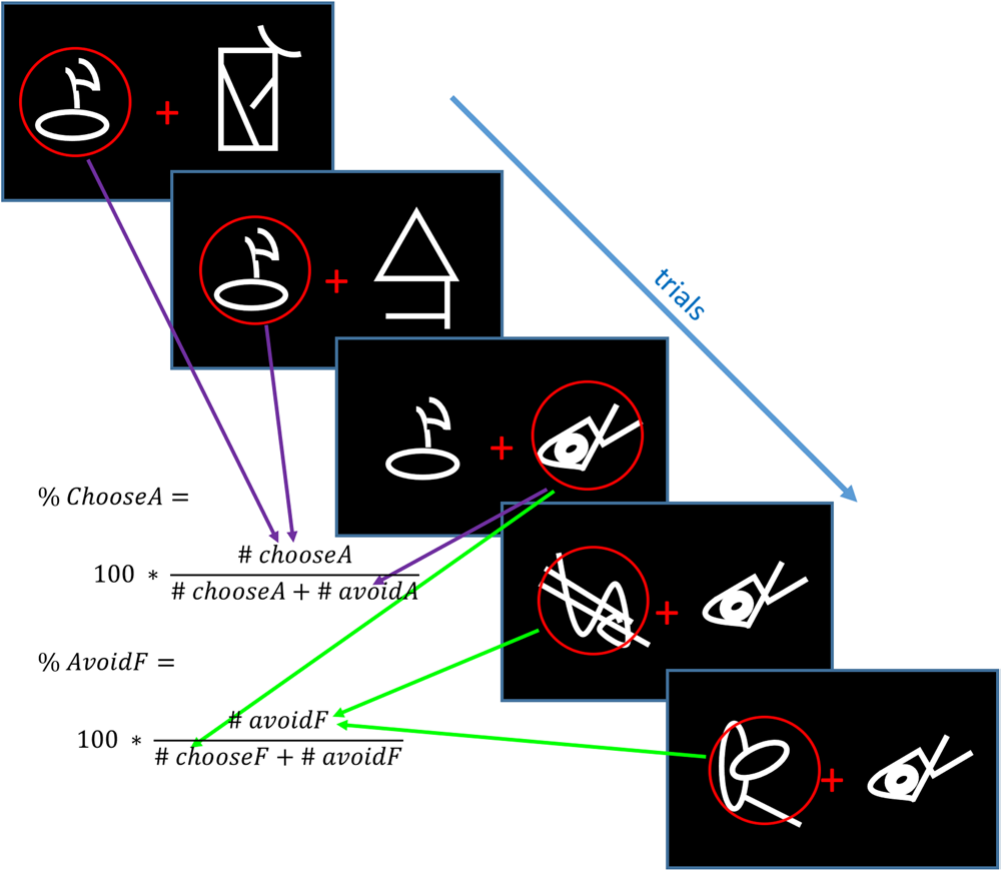
Diagram showing how Choose-A and Avoid-F were calculated in the choice phase. The same procedure was used for all symbols (representative symbols shown here).

For the choice phase, the number of times each symbol was chosen was divided by the number of times it was seen, to give percentage selections (see Fig. 3). Percentage avoidances were calculated likewise. Within-subject ANOVAs and t-tests were used on the 24-hour choice phase measures to see how levodopa affected choice performance. Cohen’s *d* and partial *η*^2^ (${\eta }_{p}^{2}$) effect sizes are reported alongside t-tests and ANOVAs, respectively. If Mauchly’s test of sphericity was significant, the Greenhouse-Geisser correction to the degrees of freedom was applied. We used SPSS v23 (IBM) for statistics. Q-Q plots were used to verify that data were approximately normal before parametric tests.

In addition to frequentists statistical analyses, we also performed Bayesian analyses in JASP^31^. Bayesian t-tests and repeated measures ANOVAs were used. Bayesian analysis compares the likelihood of the data given the null hypothesis (H_0_) to the likelihood given the experimental hypothesis (H_1_). The ratio of these two gives the Bayes Factor (BF_01_ = H_0_/H_1_) which quantifies how much more likely the data are given the null hypothesis rather than the experimental hypothesis. Please note that BF can also be reported in terms of the experimental hypothesis (i.e. BF_10_ = H_1_/H_0_), but we use the BF_01_ here due to the direction of results we found. BF of 1 suggest equal evidence for the two hypotheses, while the further the BF is from 1, the stronger the evidence for or against the null. We used the default prior of a Cauchy distribution with width 0.707 (meaning we assume there is a 50% probability of the effect size being between −0.707 and 0.707). Robustness checks with different prior widths are provided in the Supplementary Materials.

While levodopa is not prescribed based on body-weight, a previous study showed dose-dependent effects of levodopa on episodic memory consolidation when body weight was used to adjust the doses^32^. Body weight affects total absorption of levodopa, and the elimination half-life^33^, thus affecting the concentration of dopamine available in the brain. Therefore, we divided the levodopa dose (150 mg) by body weight (kg) to give the weight-adjusted doses (mg/kg) and looked for linear or polynomial regressions between this and the difference in accuracy and choices between drug and placebo conditions.

We fit two computational reinforcement learning models to the behavioural data to examine the effects on softmax choice parameters; a Q-learning^34^ model with 2 learning rates and one choice parameter, and an OpAL^35^ model with 2 learning rates and 2 choice parameters. Separate parameters were used for day 1 learning trials and day 2 testing trials. Full details are provided in Supplementary Materials.

## Results

Participants were not able to guess correctly which day they received the drugs or placebo. Twenty-nine participants provided guesses, of which 17 were correct, and a binomial test showed this was not significantly different from chance (p = 0.720).

### Learning accuracy

During learning trials, overall mean accuracy was slightly higher on the Gain pair (mean 57% accuracy, SD = 14.4) than the Loss pair (mean = 53%, SD = 11.8; Look pair mean = 52% %, SD = 14.0), although this difference was not significant (pair * drug ANOVA, pair effect: F (1, 30) = 2.508, p = 0.124, ${\eta }_{p}^{2}$ = 0.077).

### Does levodopa affect choice phase accuracy

**Figure 4:**
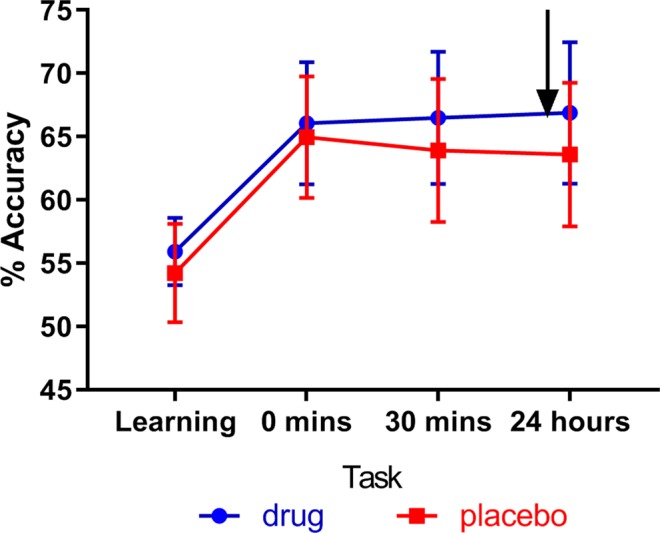
The mean % accuracy on learning and choice phases, for both conditions. The arrow shows when the drug/placebo was administered (time not to scale). There was no difference between accuracy after drug or placebo (p = 0.372, BF_01_ = 3.581; 95% confidence intervals).

The mean accuracies were much higher for the choice phases at 0 minutes, 30 minutes and 24 hours (> 65%; see Fig. 4). Performance did not change over the 3 choice tests, as shown by no significant effect of time (nor drug nor interaction) in a time * drug repeated measures ANOVA (p > 0.05; see Table 2 for statistics). As the drug/placebo was only given before the 24-hour choice phase, we used paired t-tests to look at the accuracy separately on this phase, which revealed accuracy was not affected by levodopa (t (30) = 0.906, p = 0.372, *d* = 0.163; BF_01_ = 3.581).

**Table 2:** Time * drug ANOVAs on accuracy and selections.

Effect	Time			Drug			Time * Drug		
Measure	F	p	${\eta }_{p}^{2}$	F	p	${\eta }_{p}^{2}$	F	p	${\eta }_{p}^{2}$
Accuracy	0.202	0.817	0.007	0.425	0.520	0.014	0.455	0.637	0.015
Choose-A	1.505	0.232	0.049	0.230	0.635	0.008	0.031	0.969	0.001
Choose-B	0.142	0.868	0.005	1.121	0.299	0.037	0.282	0.755	0.010
Choose-C	1.077	0.347	0.036	0.063	0.804	0.002	1.927	0.155	0.062
Choose-D	0.568	0.570	0.019	0.019	0.892	0.001	0.674	0.514	0.023
Choose-E	0.387	0.681	0.013	1.446	0.239	0.047	0.341	0.713	0.012
Choose-F	0.503	0.607	0.017	3.093	0.089	0.096	1.230	0.300	0.041

Statistical output from the two-way repeated measures ANOVAs (time * drug) on accuracy and each choice across the three choice phases. No effects or interactions were significant. df for the three columns are (2, 58), (1, 29), (2, 58).

We investigated why learning accuracy might have been so low. We found no correlations between age and learning or choice accuracy (p > 0.5; Table S4) but did find that MoCA (a measure of cognitive impairment) correlated with learning accuracy in both conditions (drug: r = 0.364, p = 0.044; placebo: r = 0.388, p = 0.031) and with choice phase accuracy only in the drug condition (drug: r > 0.47, p < 0.01; placebo r < 0.25, p > 0.2; Table S4). Importantly, while these latter correlations might suggest that levodopa is interacting with cognitive impairment to affect accuracy, the correlations were seen in the drug condition at the 0-minute and 30-minute choice phases, which occurred *before* the drug was given and therefore suggest that the drug itself had no effect. Further supporting this view, we found no correlation of MoCA with the difference in 24-hour accuracy between the two conditions (r = 0.233, p = 0.206), and no effect of including MoCA as a covariate in any accuracy analyses (p > 0.05; see Table S6).

### Positive and negative choices

**Figure 5:**
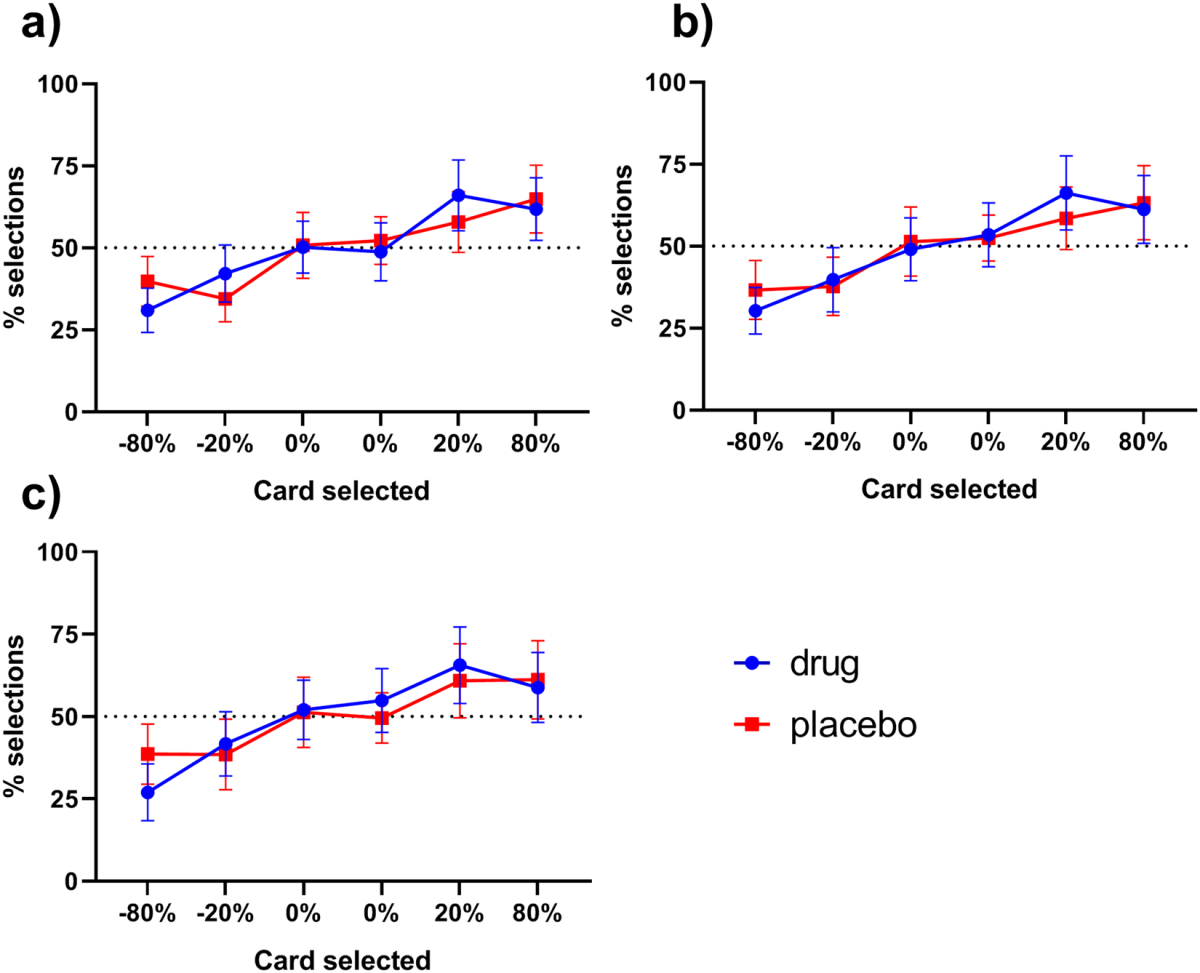
The mean percentage of choices of each symbol for both conditions (95% confidence intervals) at (**a**) 0-minutes, (**b**) 30-minutes, (**c**) 24-hours. The value of the symbol is the sum of the probability multiplied by the value of each outcome (i.e. 80% chance of loss (−1) and 20% chance of nothing (0) gives −80%). There were no significant effects of time or drug across the phases, nor any differences between drug and placebo conditions the 24-hour test (p > 0.05, BF_01_ > 1).

We divided the number of times participants chose each symbol by the number of times it was presented to give the percentage of choices of each symbol (see Fig. 3). Figure 5 shows the mean percentages of the selections of each symbol for the drug and placebo conditions at each choice phase. We looked to see whether performance changed over the three choice phases, including drug/placebo as a factor; if levodopa affected behaviour on the 24-hour choice phase there would be a time * drug interaction. No effects of time, drug or interaction were found for any choice (p > 0.05; see Table 2 for statistics).

### Does levodopa affect positive and negative choices

Paired t-tests on the 24-hour choice phase showed no significant differences in percentage of choices on drug or placebo for any of the symbols (p > 0.05; see Table 3 for statistics), suggesting that levodopa did not affect selection for any choice. Bayesian t-tests showed moderate evidence in favour of the null hypothesis (BF_01_ > 3) for all choices apart from symbol F where the evidence for the null hypothesis was anecdotal (BF_01_ = 1.301; see Table 3). This suggests that levodopa does not affect choice selection, except for the most punished symbol where the evidence is inconclusive.

**Table 3:** Frequentist and Bayesian t-tests on 24-hour choice phase.

Measure	t	p	d	95% Conf Int	BF_01_	Posterior	95% Cred Int
Accuracy	0.906	0.372	0.163	−4.135, 10.730	3.581	0.148	−0.186, 0.482
Choose-A	−0.332	0.742	−0.060	−16.919, 12.188	4.960	−0.052	−0.391, 0.281
Choose-B	0.718	0.478	0.129	−8.725, 18.187	4.115	0.115	−0.214, 0.455
Choose-C	0.878	0.387	0.158	−6.981, 17.519	3.663	0.143	−0.200, 0.494
Choose-D	0.108	0.915	0.019	−13.463, 14.968	5.192	0.019	−0.308, 0.355
Choose-E	0.454	0.653	0.082	−11.277, 17.729	4.744	0.071	−0.252, 0.415
Choose-F	−1.771	0.087	−0.318	−25.002, 1.177	1.301	−0.288	−0.642, 0.055

Statistics from frequentist and Bayesian t-tests on the accuracy and percentage of choices for each symbol at the 24-hour choice test. BF_01_ > 3 reflects moderate evidence in favour of the null hypothesis. Cohen’s d and 95% confidence intervals are presented for frequentist t-tests, and the posterior median and 95% credible intervals for the Bayesian t-tests. All error % from the Bayesian analyses were < 4 × 10^−4^.

We ran a repeated measures ANOVA to see whether levodopa affected the selection of the most rewarded and punished symbols differently (this is analogous to the ANOVAs run on choose-A and avoid-B in Frank *et al*.^1^). Looking just at the number of times the most rewarded symbol was chosen (choose-A) and the number of times the most punished symbol was avoided (avoid-F), there was no effect of medication (F (1, 30) = 0.719, p = 0.403, ${\eta }_{p}^{2}$ = 0.023) or choice (F (1, 30) = 3.058, p = 0.091, ${\eta }_{p}^{2}$ = 0.092), nor an interaction of medication and choice (F (1, 30) = 2.851, p = 0.102, ${\eta }_{p}^{2}$ = 0.087). This again suggests that levodopa did not affect expression of positive or negative reinforcement (Fig. 4; avoid-F is the inverse of choose-F) and that punishment-avoidance and reward-selection were equal in this task. A Bayesian repeated measures ANOVA found that this data was most likely under the null model (with no effects of medication, choice, or interactions; BF_M_ = 3.252) arguing against the inclusion of medication or choice in the model (BF_inclusion_ < 1).

### Additional analyses

The lack of effect here was surprising given previous studies’ findings^6,14^, so we investigated whether factors such as age, relative levodopa dose, or cognitive function could have contributed to the lack of effect.

Weight-adjusted dose did not have any significant linear or polynomial associations with the difference (between levodopa and placebo conditions) in 24-hour choice accuracy or on the difference on any of the choices (*r*^2^ < 0.017, p > 0.2; Table S2). Nor did we find any associations between 24-hour accuracy or choice behaviour and MoCA, DASS, BIS, LARS, age, or years of education (p > 0.05; see Table S3). Several participants had low MoCAs, so we included age and MoCA as covariates in the frequentist analyses reported above, which did not return any significant interactions with these covariates or produce different main effects (p > 0.05; see Tables S6 & S7).

As mentioned above, overall learning accuracy was low, so we applied post-hoc thresholding to the data, only including participants who had greater than 60% accuracy overall, or on just the Gain pair or Loss pair, or on the accuracy on the final 10 presentations of the Gain or Loss pair. This left 21 participants in the drug condition and 18 in the placebo condition; only 12 participants passed for both conditions so between-subject analyses were used. The only significant effect found was an overall effect of drug on choose-F (F (1, 37) = 5.189, p = 0.029, ${\eta }_{p}^{2}$ = 0.123). However, this does not mean that in these high-learners the drug decreased choose-F, as it was an overall effect and the drug * time interaction was not significant (F (2, 74) = 0.464, p = 0.630, ${\eta }_{p}^{2}$ = 0.012), meaning that the drug group had lower choose-F across all three choice phases, including before the drug was given, thus suggesting that levodopa did not affect choice behaviour in these high learners.

We also split participants into those who showed a negative effect of levodopa on digit span manipulation accuracy^30^, and those who did not. Including the subgrouping as a between-subject factor did not affect the results (see Table S7). This suggests that the lack of effect here was the same in those who showed effects of dopamine on the digit span, and those who did not.

We also looked at overall reaction times and found no difference between reaction times when on drug or placebo (p > 0.05; see Supplementary Materials).

### Computational Modelling

We fit two reinforcement learning models (Q-learning and OpAL model; see Supplementary Materials for model details) to the behavioural data, with separate parameters for the 24-hour choice phase, to see whether levodopa affected the choice mechanisms. As there was no feedback on the 24-hour choice phase, the only parameters that are fit to that phase are the softmax inverse temperatures, which control how strictly people rely on the learned values of the stimuli versus how random their choices are. In the OpAL model there are two softmax parameters to separately control the influence of information learned through positive and negative reinforcement.

The Q-learning model fit better than the OpAL model (lower Bayesian Information Criteria^36^; 369.7223 vs 374.6993), but its day 2 parameters did not differ between the two conditions (p > 0.2, BF_01_ > 1), nor did the day 1 parameters (p > 0.05, BF_01_ > 1; see Table 4). We also looked at the parameters from the poorer fitting OpAL model which had no significant difference between conditions either (Table S8). This suggests that levodopa does not affect the randomness of choice behaviour, or the relative influence of positive and negative learning on this.

**Table 4:** Q-learning model parameter statistics.

Measure	t	p	d	95% Conf Int	BF_01_	Posterior	95% Cred Int
*α*_+_	0.0580	0.9541	0.010	−0.342, 0.362	5.212	0.015	−0.428, 0.468
*α*_−_	−1.1782	0.2480	−0.212	−0.566, 0.146	2.776	−0.249	−0.723, 0.206
*Β*	1.4645	0.1535	0.263	−0.097, 0.619	1.990	0.306	−0.139, 0.795
*β -* day 2	1.9205	0.0643	0.345	−0.020, 0.705	1.033	0.417	−0.061, 0.915

Output from frequentist and Bayesian paired t-tests on the Q-learning model’s parameters for day 1 and day 2 data. No significant differences were found. BF_01_ > 3 reflects moderate evidence in favour of the null hypothesis. Cohen’s d and 95% confidence intervals are presented for frequentist t-tests, and the posterior median and 95% credible intervals for the Bayesian t-tests.

## Discussion

Levodopa given 24 hours after learning a reward and punishment task did not affect choice performance. This suggests that levodopa does not affect the expression of positive or negative reinforcement 24 hours after learning in older adults.

This contradicts several other studies which have found that dopamine can affect expression of reinforcement learning^4–6^. However, there are several differences between each of these studies and the current one. For example, Shiner *et al*.^4^ and Smittenaar *et al*.^5^ did not have punishments in their task, only rewards of varying probabilities. It may be that dopamine’s effects are only seen on positive reinforcement, which were missed in our task as we only had 2 stimuli that were positively reinforced (symbols A and B).

Eisenegger *et al*.^6^ used a task with positive and negative reinforcement like ours but did not have a separate ‘novel pairs’ choice phase. Instead, they looked at the performance towards the end of the learning trials and used that to assess effects on the expression of learning. While their modelling analysis suggested the effects were not due to differences in learning rates, but rather the softmax decision parameter, this was still during the learning process and thus may be quite different to processes that occur much later and do not incur feedback. It should be noted that the softmax parameter in reinforcement learning models captures how frequently participants make a ‘greedy’ selection and choose the stimuli with the highest value, rather than making an explorative choice to a lower value stimulus. Thus, it also functions like a noise parameter, and will be higher when there is more variance that the learning rate parameters cannot explain. It is possible that the true effects were not due to more random choosing but rather some unknown process during learning that was simply captured by this noise parameter.

Alternatively, perhaps our participants did not learn the task well enough for us to be able to detect differences. The average accuracy at the end of the learning trials was close to chance, though it increased on the novel pairs choice phase to levels seen in other studies^1,4,5^ (i.e. 50–80%). The poor learning may have been compounded by the inclusion of several participants with low MoCAs; MoCA correlated negatively with learning accuracy but did not reliably correlate with accuracy on the choice phases and excluding low MoCA participants did not change the pattern or significance of results. Levodopa had no effect regardless of cognitive function, but as this was not our main focus and the experiment was not set up to test this directly, this analysis was underpowered.

Additionally, the current participants were older adults (65+ years) whereas the majority of studies using this task have been on young adults^14–16,37,38^. We chose older adults as they have reduced dopaminergic activity^10^, however as dopamine receptors and transporters seem more affected by age it may be that this actually reduced the effect of the drug in our sample. Age did not correlate with accuracy or choice measures, although this may be due to the narrow age-range tested here. It is possible that levodopa may affect expression of reinforcement learning in young healthy participants while not doing so in older adults, thus different results may be found if this experiment were repeated in young adults, and if performance thresholds were applied during the learning phase.

Several other studies have combined positive and negative outcomes with a transfer task^15–17^. Our data are similar to the ‘partial information’ feedback condition from some of these studies^15,16^ with an increase in choices with increasing value. Our study gave such a task three times, with the third one occurring after drug/placebo administration. It is possible that the repeated testing in our study changed the framing of the 24-hour choice phase to more of an explicit memory task, rather than a test of implicit learning, although the lack of difference between performance across the 3 choice phases and the similarity with previous studies argues against this.

The lack of effect of levodopa on anything could also suggest that the drug simply was not having an effect. However, we used a fairly large dose (150 mg levodopa), which is as large as or larger than several other studies^11,14,18,19,39–41^. We waited 1 hour between dosing and testing, which coincides with the time to max concentration^42,43^. Although levodopa is not prescribed based on weight, higher weight (and thus larger size) decreases absorption and concentrations of levodopa^33^ and will lead to lower relative doses reaching the brain. The dopamine overdose hypothesis suggests that too high or low levels of dopamine would impair function, so the relative doses people received may affect the results. Some studies have reported dose-dependent effects, with people who had larger relative doses showing greater effects^44^. We found no such associations, linear or quadratic. Additionally, levodopa did affect the digit span in some participants (see^30^ for details), and when we looked specifically in the participants who showed that effect there was still no effect in the GainLoss task. This suggests the lack of effect was not due to the specific dosage given.

Finally, if dopamine does not affect expression of reinforcement learning, then how can we explain previous results? One possibility is that overall dopamine levels do not affect expression/retrieval, but rather that D2 receptor activation does, as suggested by Eisenegger *et al*.^6^. An alternate explanation is that previous effects were driven by consolidation. Consolidation is a mechanism often overlooked in this type of memory, but in between learning the values and retrieving them, those values must be stored for a period and protected against interference from other learning. It may be that previous effects can be explained by consolidation, as the dopamine drugs were either present during learning (and thus consolidation after learning) or given just after learning before a 1-hour delay (which would have allowed consolidation to be affected). Previous studies have suggested dopamine may affect the persistence of reinforcement learning across time^8,45^, and this is a possible avenue for future research.

## Supplementary information

Supplementary Matierals 1

## Data Availability

Data are available at the University of Bristol data repository, data.bris, at 10.5523/bris.qpqzeqc3q53m2dwczp69q3pv0^46^. Our Matlab code for the analysis is available here 10.5281/zenodo.1438407^47^, and the code for the GainLoss task is available here 10.5281/zenodo.1443384^48^.

## References

[1] Frank MJ, Seeberger LC, O’Reilly RC (2004). By carrot or by stick: cognitive reinforcement learning in parkinsonism. Science..

[2] Frank MJ, O’Reilly RC (2006). A mechanistic account of striatal dopamine function in human cognition: psychopharmacological studies with cabergoline and haloperidol. Behav. Neurosci..

[3] Cox SML (2015). Striatal D1 and D2 signaling differentially predict learning from positive and negative outcomes. Neuroimage.

[4] Shiner T (2012). Dopamine and performance in a reinforcement learning task: evidence from Parkinson’s disease. Brain.

[5] Smittenaar P (2012). Decomposing effects of dopaminergic medication in Parkinson’s disease on probabilistic action selection - learning or performance?. Eur. J. Neurosci..

[6] Eisenegger, C. *et al*. Role of Dopamine D2 Receptors in Human Reinforcement Learning. Neuropsychopharmacology 1–10, 10.1038/npp.2014.84 (2014). 10.1038/npp.2014.84 PMC4138746 24713613

[7] Vo A (2014). Dopaminergic medication impairs feedback- based stimulus-response learning but not response selection in Parkinson’s disease. Front. Hum. Neurosci..

[8] Grogan JP (2017). Effects of dopamine on reinforcement learning and consolidation in Parkinson’s disease. Elife.

[9] Cools R (2006). Dopaminergic modulation of cognitive function-implications for L-DOPA treatment in Parkinson’s disease. Neurosci. Biobehav. Rev..

[10] Karrer, T. M., Josef, A. K., Mata, R., Morris, E. D. & Samanez-Larkin, G. R. Reduced dopamine receptors and transporters but not synthesis capacity in normal aging adults: a meta-analysis. *Neurobiol. Aging*, 10.1016/j.neurobiolaging.2017.05.006 (2017). 10.1016/j.neurobiolaging.2017.05.006 PMC5645072 28599217

[11] Vo A, Seergobin KN, MacDonald PA (2017). Effects of levodopa on stimulus-response learning versus response selection in healthy young adults. Behav. Brain Res..

[12] Gallant H, Vo A, Seergobin KN, Macdonald PA (2016). Pramipexole Impairs Stimulus-Response Learning in Healthy Young Adults. Front. Neurosci..

[13] Cools R, Barker RA, Sahakian BJ, Robbins TW (2001). Enhanced or Impaired Cognitive Function in Parkinson’s Disease as a Function of Dopaminergic Medication and Task Demands. Cereb. cortex.

[14] Pessiglione M, Seymour B, Flandin G, Dolan RJ, Frith CD (2006). Dopamine-dependent prediction errors underpin reward-seeking behaviour in humans. Nature.

[15] Palminteri S, Khamassi M, Joffily M, Coricelli G (2015). Contextual modulation of value signals in reward and punishment learning. Nat. Commun..

[16] Palminteri S, Kilford EJ, Coricelli G, Blakemore SJ (2016). The Computational Development of Reinforcement Learning during Adolescence. PLoS Comput. Biol..

[17] Palminteri S (2012). Critical Roles for Anterior Insula and Dorsal Striatum in Punishment-Based Avoidance Learning. Neuron.

[18] Chowdhury R (2013). Dopamine restores reward prediction errors in old age. Nat. Neurosci..

[19] Guitart-Masip M (2014). Differential, but not opponent, effects of L -DOPA and citalopram on action learning with reward and punishment. Psychopharmacology (Berl)..

[20] Pelli DG (1997). The VideoToolbox software for visual psychophysics: transforming numbers into movies. Spat. Vis..

[21] Brainard DH (1997). The Psychophysics Toolbox. Spat. Vis..

[22] Kleiner M (2007). What’s new in Psychtoolbox-3? A free cross-platform toolkit for Psychophysics with Matlab & GNU/Octave. Perception.

[23] Wechsler, D. *Wechsler Adult Intelligence Scale—Fourth Edition* (WAIS–IV). (NCS Pearson, 2008).

[24] Ellis BW (1981). The St. Mary’s Hospital sleep questionnaire: a study of reliability. Sleep.

[25] Nasreddine ZS (2005). The Montreal Cognitive Assessment, MoCA: A brief screening tool for mild cognitive impairment. J. Am. Geriatr. Soc..

[26] Patton JH, Stanford MS, Barratt ES (1995). Factor structure of the Barratt impulsiveness scale. J. Clin. Psychol..

[27] Sockeel P (2006). The Lille apathy rating scale (LARS), a new instrument for detecting and quantifying apathy: validation in Parkinson’s disease. J. Neurol. Neurosurg. Psychiatry.

[28] Lovibond, S. H. & Lovibond, P. F. *Manual for the Depression Anxiety Stress Scales.* (Sydney: Psychology Foundation, 1995).

[29] Pacini R, Epstein S (1999). The relation of rational and experiential information processing styles to personality, basic beliefs, and the ratio-bias phenomenon. J. Pers. Soc. Psychol..

[30] Grogan, J. P., Knight, L. E., Smith, L. & Izagirre, N. I. Effects of Parkinson’ s disease and dopamine on digit span measures of working memory. *Psychopharmacology (Berl)*, 10.1007/s00213-018-5058-6 (2018). 10.1007/s00213-018-5058-6 PMC6267128 30315362

[31] JASP (Version 0.8.0.1)[Computer software] (2018).

[32] Chowdhury R, Guitart-Masip M, Bunzeck N, Dolan RJ, Düzel E (2012). Dopamine Modulates Episodic Memory Persistence in Old Age. J. Neurosci..

[33] Zappia M (2002). Body weight influences pharmacokinetics of levodopa in Parkinson’s disease. Clin.Neuropharmacol..

[34] Sutton, R. S. & Barto, A. G. *Reinforcement Learning: An Introduction* (MIT Press, 1998).

[35] Collins AGE, Frank MJ (2014). Opponent Actor Learning (OpAL): modeling interactive effects of striatal dopamine on reinforcement learning and choice incentive. Psychol. Rev..

[36] Schwarz G (1978). Estimating the Dimension of a Model. Ann. Stat..

[37] Lefebvre G, Lebreton M, Meyniel F, Bourgeois-Gironde S, Palminteri S (2017). Behavioural and neural characterization of optimistic reinforcement learning. Nat. Hum. Behav..

[38] Vinckier F (2015). Confidence and psychosis: a neuro-computational account of contingency learning disruption by NMDA blockade. Mol. Psychiatry.

[39] Rutledge RB, Skandali N, Dayan P, Dolan RJ (2015). Dopaminergic Modulation of Decision Making and Subjective Well-Being. J. Neurosci..

[40] Wunderlich K, Smittenaar P, Dolan RJ (2012). Dopamine enhances model-based over model-free choice behavior. Neuron.

[41] Vo A, Seergobin KN, Morrow SA, Macdonald PA (2016). Levodopa impairs probabilistic reversal learning in healthy young adults. Psychopharmacology (Berl)..

[42] Chaná P, Fierro A, Reyes-Parada M, Sáez-Briones P (2003). Pharmacokinetic comparison of Sinemet and Grifoparkin (levodopa/carbidopa 250/25 mg) in Parkinson s disease: a single dose study. Rev. Med. Chil..

[43] Nishikawa N (2012). Coadministration of Domperidone Increases Plasma Levodopa Concentration in Patients With Parkinson Disease. Clin. Neuropharmacol..

[44] Chowdhury R (2013). Supplementary: Dopamine restores reward prediction errors in old age. Nat. Neurosci..

[45] Coulthard EJ (2012). Distinct roles of dopamine and subthalamic nucleus in learning and probabilistic decision making. Brain.

[46] Grogan, J. P., Isotalus, H. K. & Coulthard, E. J. *Gain Loss Data Release*, 10.5523/bris.qpqzeqc3q53m2dwczp69q3pv0 (2018).

[47] Grogan, J. P. *Gain Loss Analysis*, 10.5281/zenodo.1438407 (2018).

[48] Grogan, J. P. & Isotalus, H. K. *Gain Loss Task*, 10.5281/zenodo.1443384 (2018).

