# Supplementary material for: Levodopa does not affect expression of reinforcement learning in older adults
Source: Sci Rep. 2019 Apr 23;9:6349. doi: 10.1038/s41598-019-42904-5 (PMC6478852; doi:10.1038/s41598-019-42904-5)
Supplement: Supplementary file 1 — Supplementary Matierals 1 [file 41598_2019_42904_MOESM1_ESM.docx]

Supplementary Materials 1: Levodopa does not affect expression of reinforcement learning in older adults

Grogan, J.P.^1*^, Isotalus, H.K.^1^, Howat, A.^1^, Irigoras Izagirre, N.^1^, Knight, L.E.^2^, & Coulthard, E.J.^1,3*^

1. University of Bristol, Bristol, UK.
2. University Hospitals Bristol, Bristol, UK.
3. North Bristol NHS Trust, Bristol, UK.

*Correspondence to: [john.grogan@bristol.ac.uk](mailto:john.grogan@bristol.ac.uk) or [Elizabeth.coulthard@bristol.ac.uk](mailto:Elizabeth.coulthard@bristol.ac.uk)

# Learning


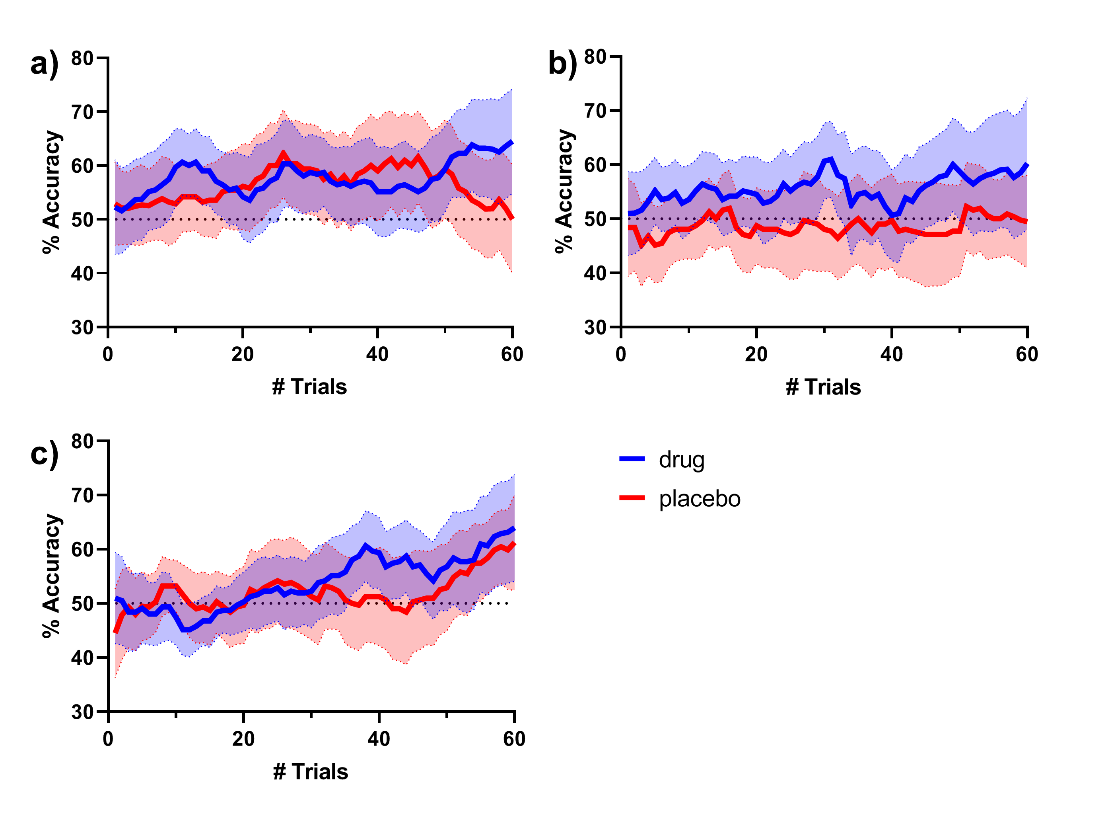


Figure S1. Moving mean of the % of optimal choices in the a) Gain, b) Look, and c) Loss pair across the learning trials for both conditions (95% CI). For the Look pair, symbol C was classed as the optimal response.

# Individual data

Here are figures showing the individual data presented in the figures in the main text.


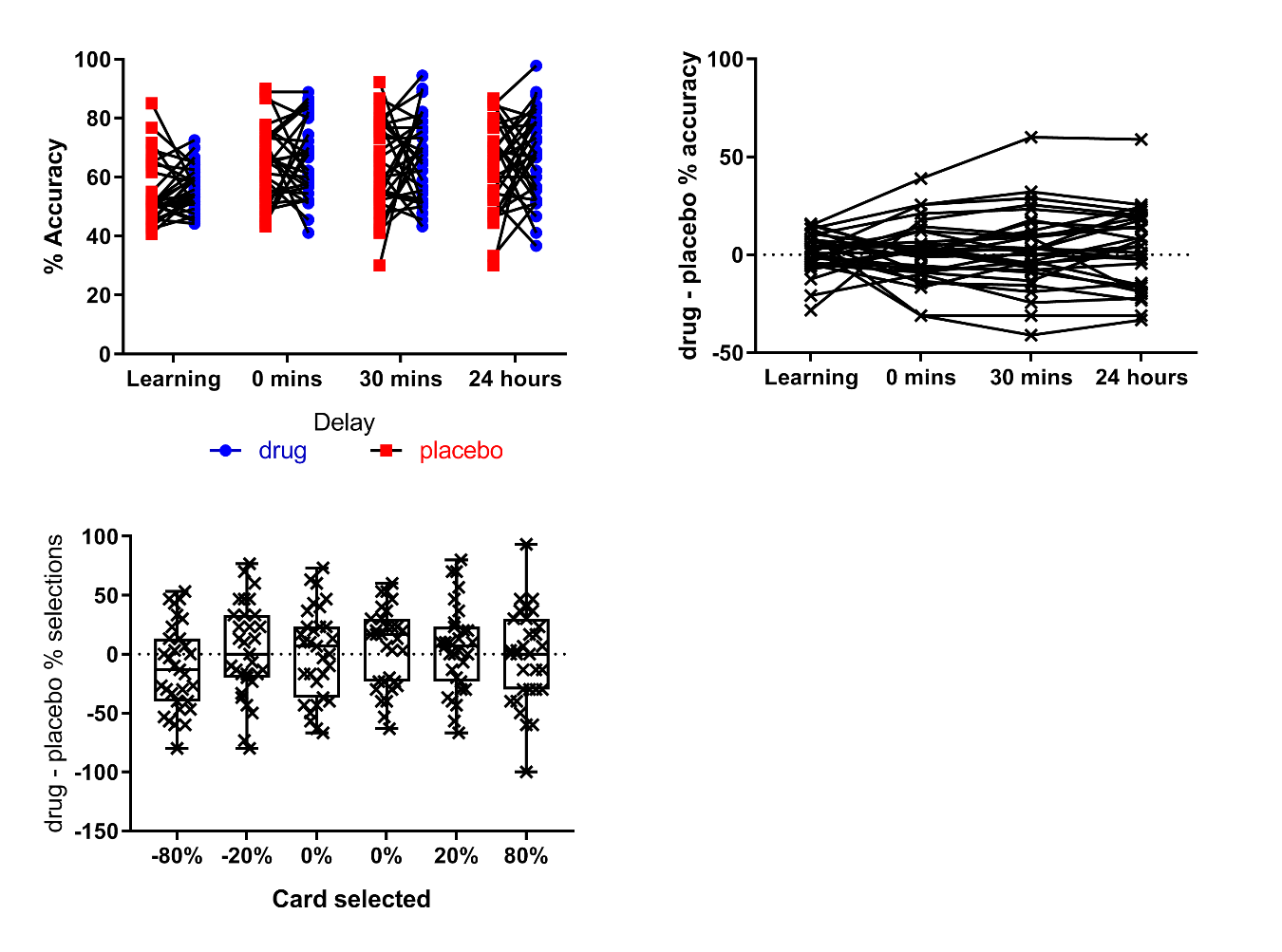


Figure S2. Individual accuracies for drug and placebo conditions for learning and tests (a), the difference between these conditions (b), and the difference in % selections at the 24-hour test (c).


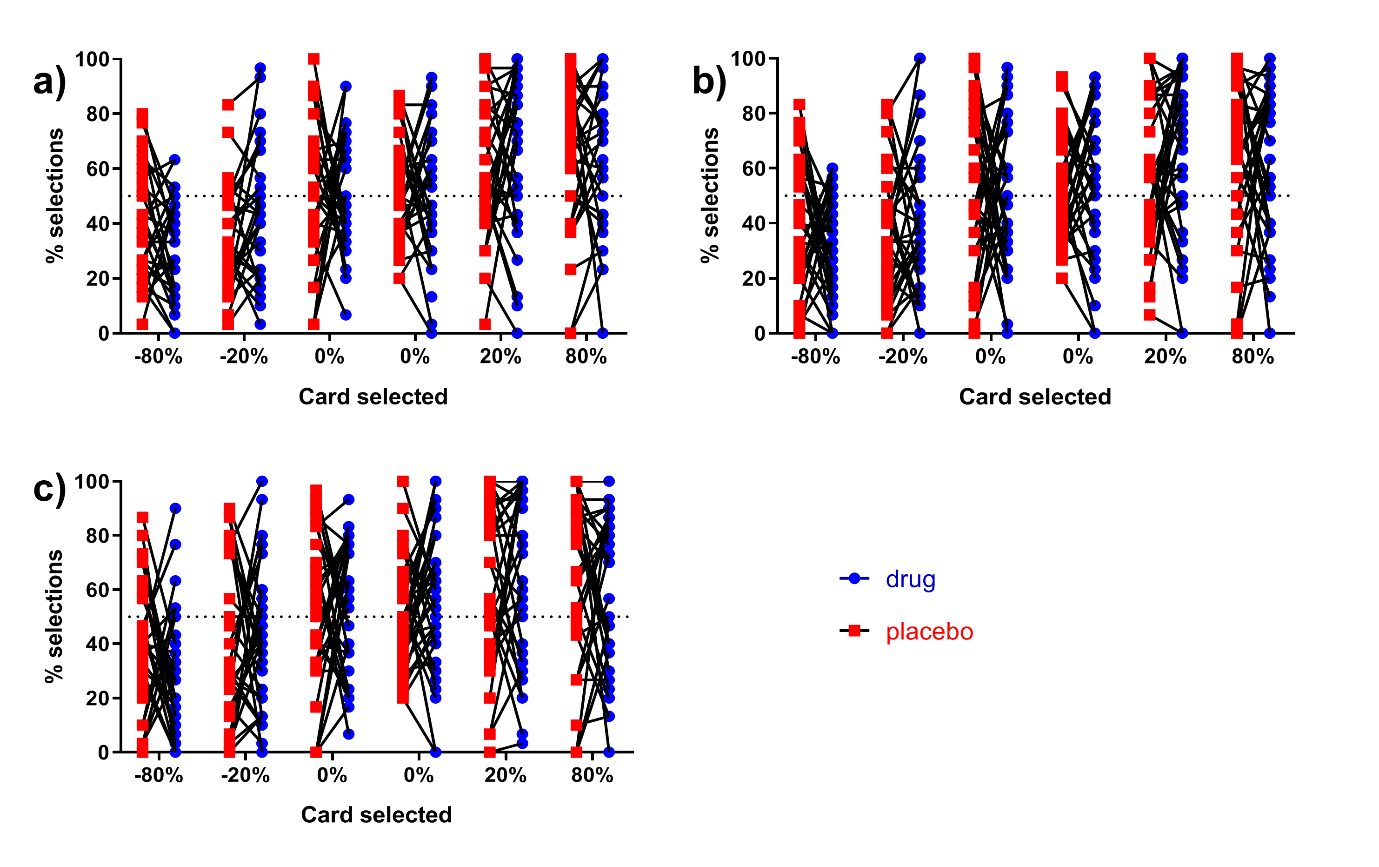


Figure S3. The % of choices made by each participant in each condition at each of the three tests: a) 0 minutes, b) 30 minutes, c) 24 hours.

# Summary statistics

Here are tables giving the means and standard deviations for each figure and statistical test in the manuscript.

Table S1. Summary statistics (mean, standard deviation) for each figure and statistic presented in the manuscript. Accuracy data relate to Fig. 4, and ‘choose’ data to Fig. 5. All variables had 31 participants’ data, except for the 0-min test which had 1 missing datum due to computer error.

|  |  | Drug | | Placebo | |
| --- | --- | --- | --- | --- | --- |
| Test | Measure | Mean | SD | Mean | SD |
| Learning | Accuracy | 55.91 | 7.26 | 54.22 | 10.60 |
| 0-min test | Accuracy | 66.06 | 13.17 | 64.96 | 12.84 |
|  | Choose-A | 61.83 | 26.09 | 64.89 | 27.65 |
|  | Choose-B | 66.02 | 29.30 | 57.89 | 24.81 |
|  | Choose-C | 48.82 | 24.09 | 52.22 | 19.37 |
|  | Choose-D | 50.22 | 21.62 | 50.78 | 26.92 |
|  | Choose-E | 42.15 | 23.77 | 34.44 | 18.72 |
|  | Choose-F | 30.97 | 18.44 | 39.78 | 20.30 |
| 30-min test | Accuracy | 66.49 | 14.27 | 63.91 | 15.40 |
|  | Choose-A | 61.18 | 28.13 | 63.23 | 30.81 |
|  | Choose-B | 66.24 | 30.81 | 58.49 | 26.05 |
|  | Choose-C | 53.44 | 26.59 | 52.47 | 19.05 |
|  | Choose-D | 49.03 | 26.15 | 51.40 | 27.72 |
|  | Choose-E | 39.78 | 26.68 | 37.74 | 24.27 |
|  | Choose-F | 30.32 | 19.31 | 36.67 | 24.34 |
| 24-hour test | Accuracy | 66.88 | 15.23 | 63.58 | 15.48 |
|  | Choose-A | 58.81 | 28.99 | 61.18 | 32.42 |
|  | Choose-B | 65.59 | 31.77 | 60.86 | 30.65 |
|  | Choose-C | 54.84 | 26.34 | 49.57 | 20.81 |
|  | Choose-D | 52.04 | 24.51 | 51.29 | 29.17 |
|  | Choose-E | 41.72 | 26.64 | 38.49 | 29.29 |
|  | Choose-F | 26.99 | 23.53 | 38.60 | 24.93 |

# Dose-Dependent Relationships

We looked for linear or quadratic relationships between relative dose and performance on the GainLoss task at the 24-hour test. We divided the levodopa dose (150mg) by participants’ weight in kg to give relative dose (mg/kg). Weight has been shown to affect the absorption and elimination of levodopa (Zappia et al., 2002), and quadratic relationships have been reported in other studies, in line with the levodopa overdose hypothesis (Cools, 2006).

We looked for linear and quadratic relationships between relative dose and the difference on three measures (accuracy, choose-A, choose-F) between drug and placebo conditions at the 24-hour test. There were no quadratic or linear relationships found (p > .4, see Table S2 for statistics). Fig. S3 shows a scatter plot of relative doses against each measure difference, with the best fitting line (linear in each case) drawn with 95% confidence intervals; in each case this best fitting line was not significantly different to a horizontal line.


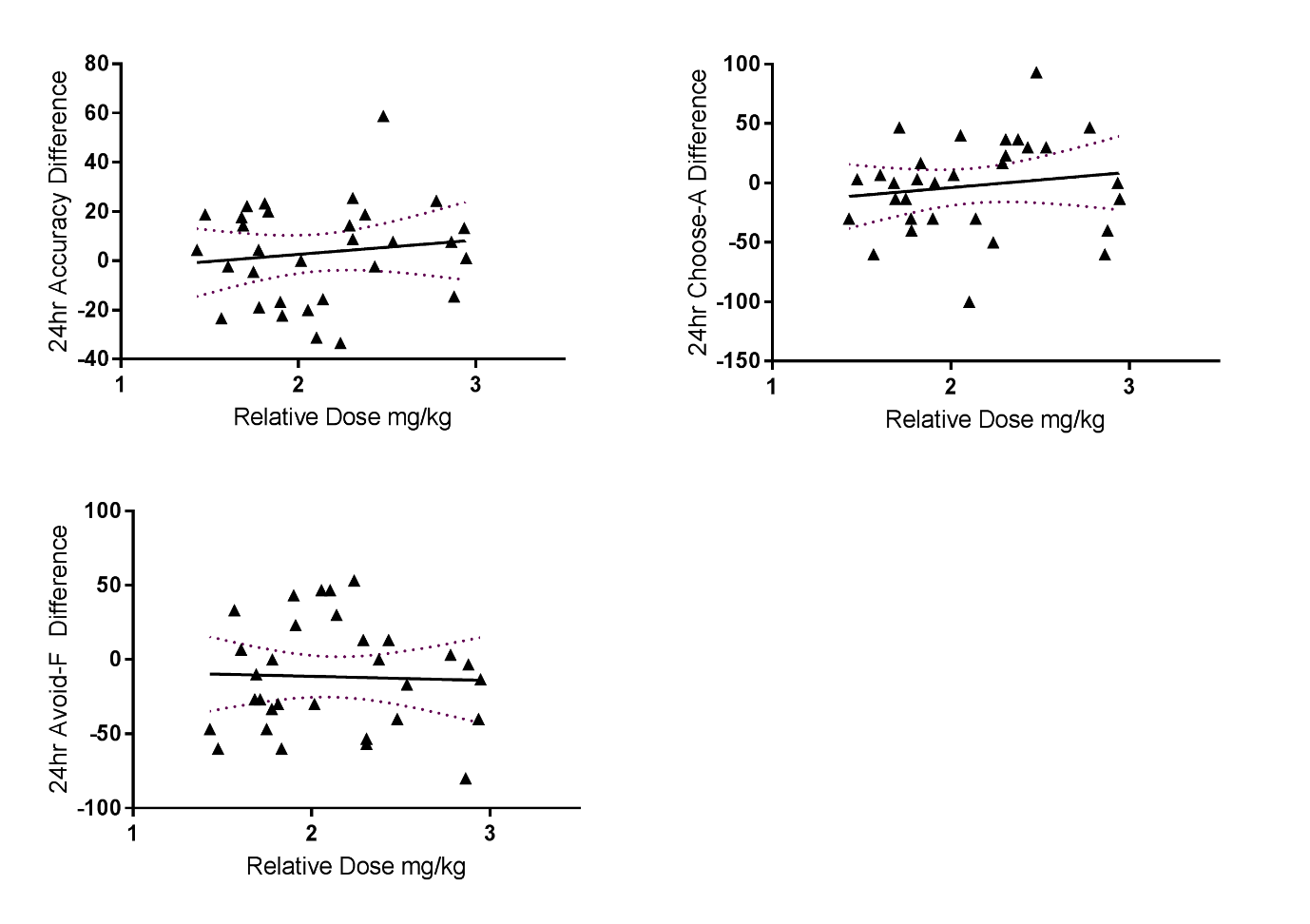


Figure S4. Relative levodopa dose (mg/kg) versus the difference (drug – placebo) in accuracy, choose-A, and choose-F at 24 hours. The best fitting line is plotted with 95% confidence intervals. None of the lines were significantly different to a flat line.

Table S2. Statistics from linear and quadratic regressions of relative levodopa dose (mg/kg) and the difference in performance measures between 24-hour drug and placebo tests.

| Measure | Relationship | β (standardised) | t | p |
| --- | --- | --- | --- | --- |
| 24hr accuracy difference | Linear | 0.130 | 0.706 | .486 |
| 24hr accuracy difference | Quadratic | 0.139 | 0.758 | .455 |
| 24hr choose-A difference | Linear | 0.148 | 0.806 | .427 |
| 24hr choose-A difference | Quadratic | 0.125 | 0.676 | .504 |
| 24hr choose-F difference | Linear | -0.035 | -0.188 | .852 |
| 24hr choose-F difference | Quadratic | -0.070 | -0.376 | .709 |

# Other Relationships

We also looked for linear relationships with MoCA, DASS, BIS and LARS. None were significant (see Table S3 and Fig. S4).


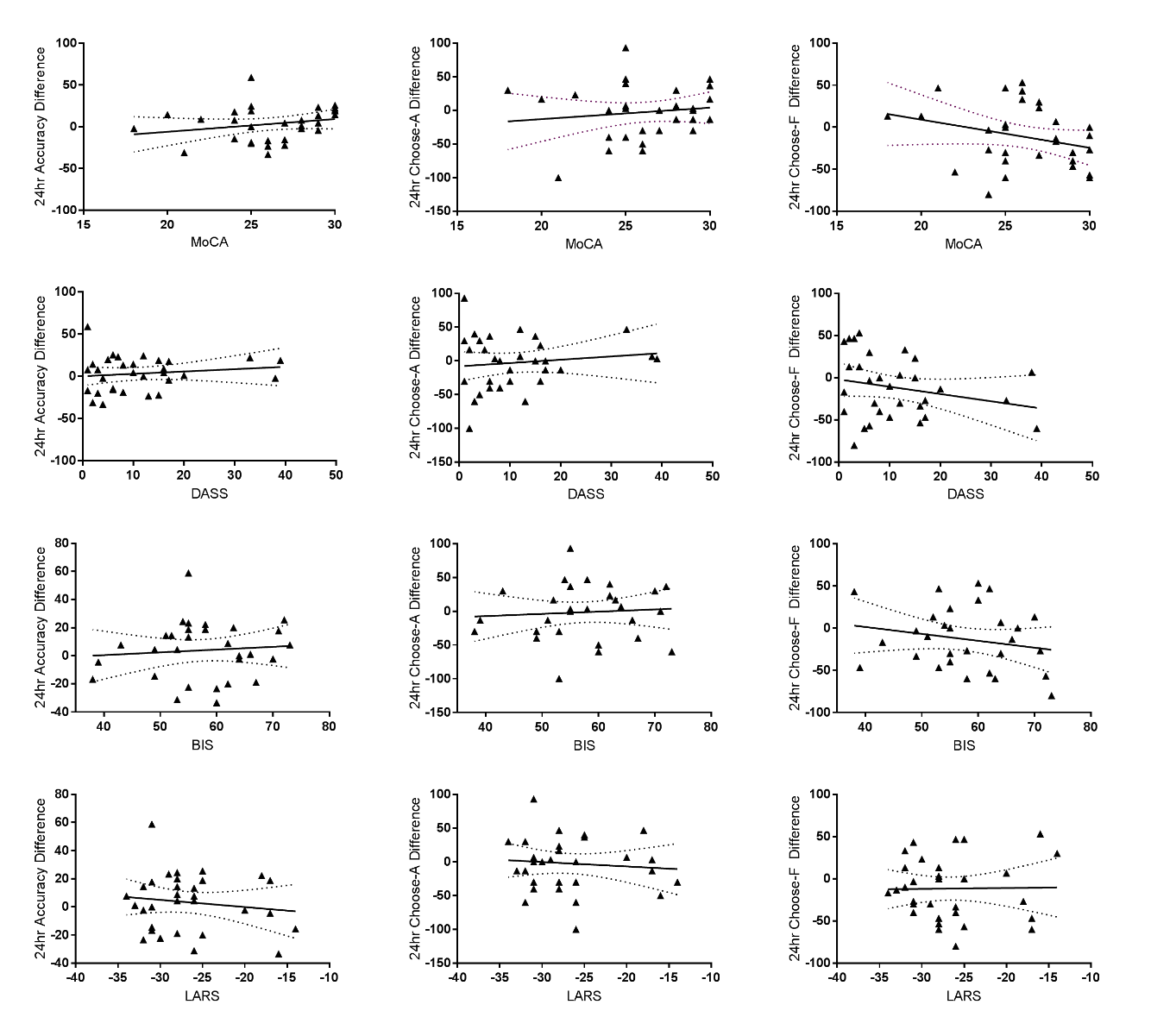


Figure S5. Scatter plots showing relationships between questionnaires and the difference between drug and placebo measures at 24 hours. The left column shows the difference between 24-hour accuracy, the middle column choose-A difference, and the right column choose-F difference. The rows show (in order): MoCA, DASS, BIS, and LARS. The best fitting line is plotted with 95% confidence intervals. None of the lines were significantly different to a flat line.

Table S3. Statistics from the linear regressions of MoCA and the difference in performance measures between 24-hour drug and placebo tests.

| Measure | IV | β (standardised) | t | p |
| --- | --- | --- | --- | --- |
| 24hr accuracy difference | MoCA | 0.233 | 1.293 | .206 |
| 24hr choose-A difference | MoCA | 0.133 | 0.722 | .476 |
| 24hr choose-F difference | MoCA | -0.285 | -1.599 | .121 |
| 24hr accuracy difference | DASS | 0.139 | 0.754 | .457 |
| 24hr choose-A difference | DASS | 0.125 | 0.677 | .504 |
| 24hr choose-F difference | DASS | -0.238 | -1.322 | .196 |
| 24hr accuracy difference | BIS | 0.087 | 0.465 | .646 |
| 24hr choose-A difference | BIS | 0.074 | 0.392 | .698 |
| 24hr choose-F difference | BIS | -0.203 | -1.098 | .282 |
| 24hr accuracy difference | LARS | -0.137 | -0.745 | .462 |
| 24hr choose-A difference | LARS | -0.090 | -0.487 | .630 |
| 24hr choose-F difference | LARS | 0.015 | 0.082 | .935 |

Table S4. The outputs from Pearson’s correlations between MoCA and age and accuracy measures across learning and testing phases. * = p < .05, ** = p < .01, *** = p < .001.

| Measure | Condition | IV | r | p |
| --- | --- | --- | --- | --- |
| Learning accuracy | Drug | MoCA | .364 | .044* |
| Learning accuracy | Placebo | MoCA | .388 | .031* |
| 0-min test accuracy | Drug | MoCA | .599 | <.001*** |
| 0-min test accuracy | Placebo | MoCA | .234 | .214 |
| 30-min test accuracy | Drug | MoCA | .471 | .008** |
| 30-min test accuracy | Placebo | MoCA | .179 | .335 |
| 24-hour test accuracy | Drug | MoCA | .489 | .005** |
| 24-hour test accuracy | Placebo | MoCA | .176 | .345 |
| Learning accuracy | Drug | Age | -.344 | .058 |
| Learning accuracy | Placebo | Age | -.277 | .131 |
| 0-min test accuracy | Drug | Age | -.129 | .490 |
| 0-min test accuracy | Placebo | Age | -.089 | .639 |
| 30-min test accuracy | Drug | Age | -.183 | .326 |
| 30-min test accuracy | Placebo | Age | -.103 | .582 |
| 24-hour test accuracy | Drug | Age | -.108 | .565 |
| 24-hour test accuracy | Placebo | Age | -.101 | .589 |

We included MoCA as a covariate in the analyses presented in the main text, which revealed no interactions of MoCA with condition for accuracy or any of the choices and did not alter the reported results (see Table S5).

Table S5. The output from within-subject one-way ANOVAs with a between-subject factor of MoCA, on 24-hour test measures. No effects were significant.

| Measure | Effect | F | p | $\boldsymbol{\eta}_{\boldsymbol{p}}^{\boldsymbol{2}}$ |
| --- | --- | --- | --- | --- |
| Accuracy | Drug | 1.387 | .248 | .046 |
| Accuracy | Drug * MoCA | 1.671 | .206 | .054 |
| Choose-A | Drug | 0.571 | .456 | .019 |
| Choose-A | Drug * MoCA | 0.521 | .476 | .018 |
| Choose-F | Drug | 1.898 | .179 | .061 |
| Choose-F | Drug * MoCA | 2.555 | .121 | .081 |

We included age as a covariate in the main analyses, which found no effects or interactions of age (Table S6).

Table S6. The output from within-subject one-way ANOVAs with a between-subject factor of age, on 24-hour test measures. No effects were significant.

| Measure | Effect | F | p | $\boldsymbol{\eta}_{\boldsymbol{p}}^{\boldsymbol{2}}$ |
| --- | --- | --- | --- | --- |
| Accuracy | Drug | 0.012 | .913 | .000 |
| Accuracy | Drug * Age | 0.000 | .984 | .000 |
| Choose-A | Drug | 0.662 | .423 | .022 |
| Choose-A | Drug * Age | 0.615 | .439 | .021 |
| Choose-F | Drug | 3.107 | .089 | .097 |
| Choose-F | Drug * Age | 2.515 | .124 | .080 |

# subgroups

We split participants into two groups: those who had a negative effect of levodopa on digit span manipulation accuracy (22/30; 1 missing digit span) (Grogan et al., 2018), and those who did not (8/30), to see whether different effects were seen in the group who did have an effect of levodopa on working memory. We included this subgrouping as a between-subject factor in the main analyses which did not give any significant effects or change the pattern of results.

Table S7. The output from within-subject one-way ANOVAs with a between-subject factor of Digit Span Subgroup, on 24-hour test measures. No effects were significant.

| Measure | Effect | F | p | $\boldsymbol{\eta}_{\boldsymbol{p}}^{\boldsymbol{2}}$ |
| --- | --- | --- | --- | --- |
| Accuracy | Drug | 1.608 | .215 | .054 |
| Accuracy | Drug * Subgroup | 1.866 | .183 | .062 |
| Choose-A | Drug | 0.710 | .791 | .003 |
| Choose-A | Drug * Subgroup | 0.221 | .642 | .008 |
| Choose-F | Drug | 3.525 | .071 | .112 |
| Choose-F | Drug * Subgroup | 0.436 | .515 | .015 |

# Reaction Times

The mean reaction times at the 24-hour test were 1648ms (SD=485ms) for the drug condition and 1764ms (SD=631ms) for the placebo condition. Due to a lognormal distribution of reaction times, we compared the mean log reaction times at the 24-hour test between the two conditions and found no significant difference (t (30) = -1.0412, p = .306, *d* = -0.187, BF_01_ = 3.181). This suggests overall reaction time was not affected by levodopa.

# Modelling

We fit computational reinforcement learning models to the data to see which model best captured the data and whether the choice parameters were affected by levodopa. We fit a Q-learning model with two learning rates (for positive and negative reinforcement) and one softmax inverse temperature parameter. In this model, the value of each stimulus *i*, *Q(i)*, on trial *t* is updated based on the reward prediction error:

$$\delta=r- Q_{t}(i)$$

where *r* is the reward received on trial *t* and *Q_t_(i)* is the reward expected. This then updates the Q values:

$$\left\{ \begin{aligned} Q_{t+1}(i)= Q_{t}(i)+ \alpha_{+}\delta\delta>0 \\ Q_{t+1}(i)= Q_{t}(i)+ \alpha_{-}\delta\delta<0 \end{aligned} \right.$$

with positive and negative learning rates (*α_+_* and *α_-_*) used for positive and negative feedback, respectively. These values are compared using the softmax equation:

$$P\left( i \right)=\frac{e^{\beta Q_{t}(i)}}{\sum_{i=1}^{n} e^{\beta Q_{t}(i)}}$$

where *β* controls how deterministic or random the action selection is.

We also fit the OpAL model (Collins & Frank, 2014) which has different learning rates for Go and NoGo pathways in the basal ganglia:

$$G_{t+1}\left( i \right)= G_{t}\left( i \right)+ \alpha_{G}\delta G_{t}$$

$$N_{t+1}\left( i \right)= N_{t}\left( i \right)- \alpha_{N}\delta N_{t}$$

which are both updated on every trial, using positive and negative learning rates *α_G_* and *α_N_*. The softmax equation is:

$$P\left( i \right)= \frac{e^{\beta_{G}G_{t}\left( i \right)-\beta_{N}N_{t}(i)}}{\sum_{i=1}^{n} e^{\beta_{G}G_{t}\left( i \right)-\beta_{N}N_{t}(i)}}$$

where *β_G_* and *β_N_* are the choice parameters for the Go and NoGo pathway, respectively, and control how much each contributes to the action selection, and therefore the bias towards Go and NoGo.

The fitting included the learning trials and the 24-hour testing trials (as there were no effects of time on performance in the choice tests, we did not include the 0-minute and 30-minute tests here); separate choice parameters were used for the 24-hour testing trials. The models were fit to the two conditions separately, using the maximum likelihood method with Matlab’s fminsearch function with 100 sets of randomly generated parameters. ‘Gains’ were coded as +1, ‘Losses’ as -1, and ‘Looks’ and ‘Nothings’ as 0. The fits were compared using the Bayesian Information Criterion (Schwarz, 1978). To test the model fitting procedure, we simulated both models 62 times (the same number of sessions as we had real data for) with randomly chosen parameters, and fit both models to both simulations using the same procedure described above. The mean BIC was lower for the correct models (176.6532 and 94.3935 for Q-learning and OpAL simulations) than incorrect models (221.3828 and 217.9810) and identified the correct model 98% of the time.

The mean BIC was lower for the Q-learning model than the OpAL model (369.7223 vs 374.6993), suggesting a better fit. The day 1 learning rates and choice parameters did not differ between the two conditions, nor did the day 2 choice parameters (Table 4 in main text).

As the Q-learning model only had 1 parameter for the choice phase, we also analysed the OpAL model’s parameters to see whether the more complex choice rule there identified any differences in parameters for the two conditions. We found no differences between parameters for the two conditions (Table S8).

Table S8. Output from frequentist and Bayesian paired t-tests on the OpAL model’s parameters for day 1 and day 2 data. No significant differences were found. BF_01_ > 3 reflects moderate evidence in favour of the null hypothesis. Cohen’s d and 95% confidence intervals are presented for frequentist t-tests, and the posterior median and 95% credible intervals for the Bayesian t-tests.

| Measure | t | p | d | 95% Conf Int | BF_01_ | Posterior | 95% Cred Int |
| --- | --- | --- | --- | --- | --- | --- | --- |
| *α_G_* | 1.887 | .069 | .3389 | -.026, .698 | 1.089 | .405 | -.063, .906 |
| *α_N_* | 0.759 | .454 | .1362 | -.219,.489 | 4.005 | .163 | -.304, .627 |
| *β_G_* | 0.725 | .474 | .1303 | -.224, .483 | 4.096 | .154 | -.298, .613 |
| *β_N_* | -0.408 | .686 | -.0732 | -.425, .280 | 4.833 | -.084 | -.552, .357 |
| *β_G_ -* day 2 | 0.880 | .386 | .1581 | -.198, .511 | 3.658 | .191 | -.269, .662 |
| *β_N_ -* day 2 | 0.682 | .501 | .1225 | -.232, .475 | 4.212 | .140 | -.314, .609 |

# Bayesian Analysis

We used Bayesian versions of frequentist statistical tests to be able to assess whether the data supported the null hypothesis, or simply were inconclusive. We used JASP software to do this. The results of the tests are presented in the main text, and here we present the plots showing the prior and posterior densities, robustness checks and sequential analyses for the 24-hour accuracy and choices.

## 24hr Accuracy

##### Prior and Posterior


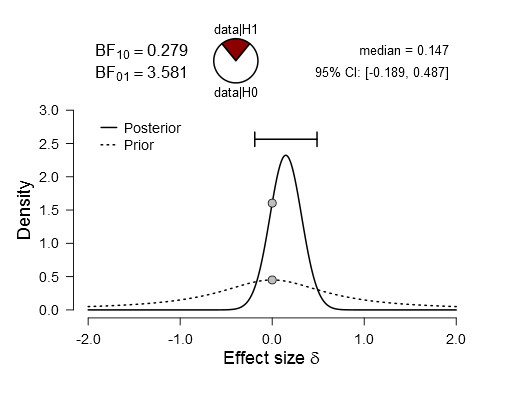


##### Bayes Factor Robustness Check


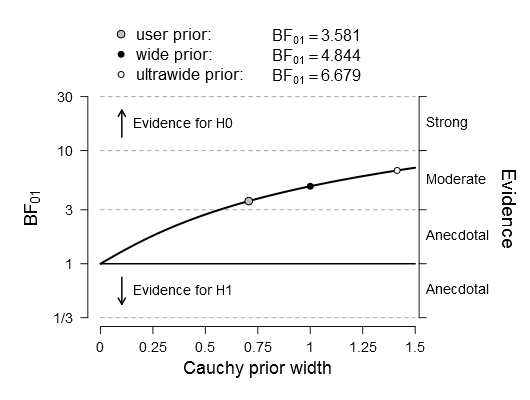


##### Sequential Analysis


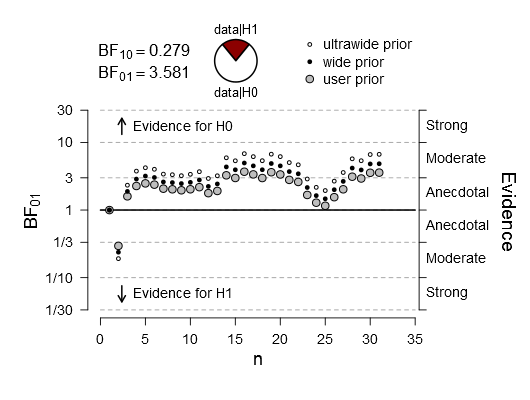


## Choose A

##### Prior and Posterior


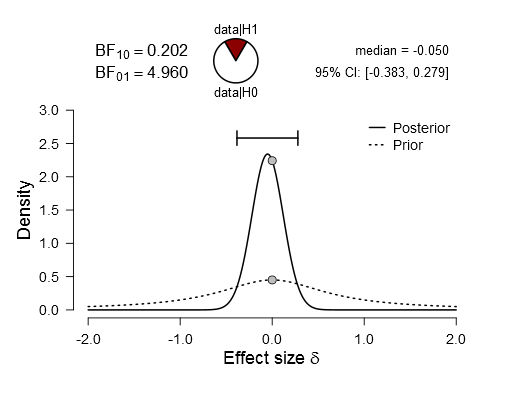


##### Bayes Factor Robustness Check


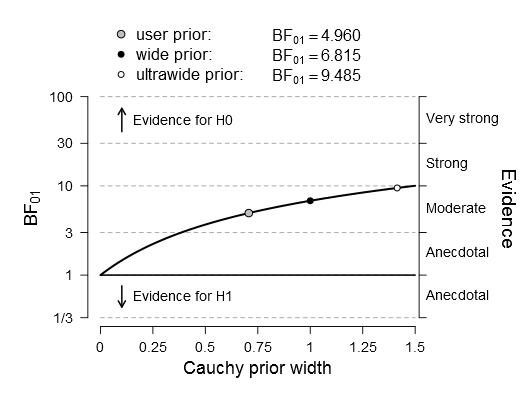


##### Sequential Analysis


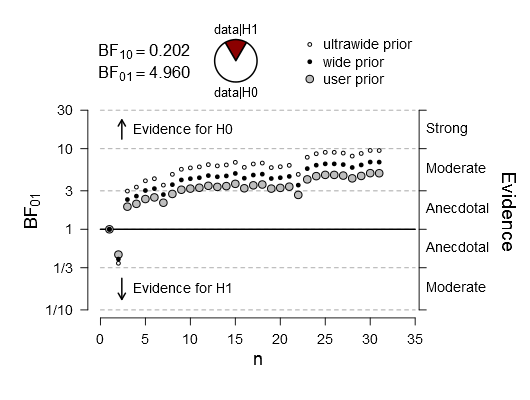


## Choose B

##### Prior and Posterior


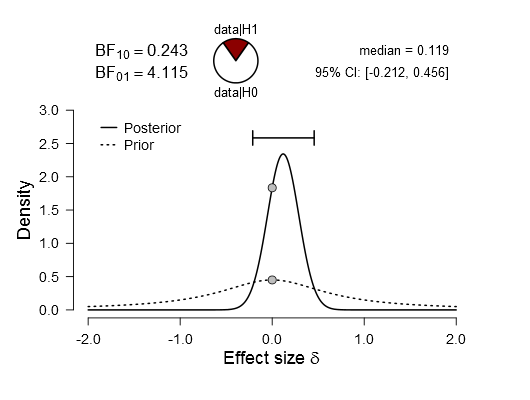


##### Bayes Factor Robustness Check


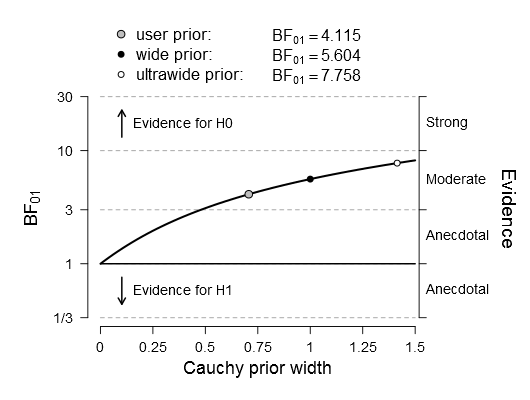


##### Sequential Analysis


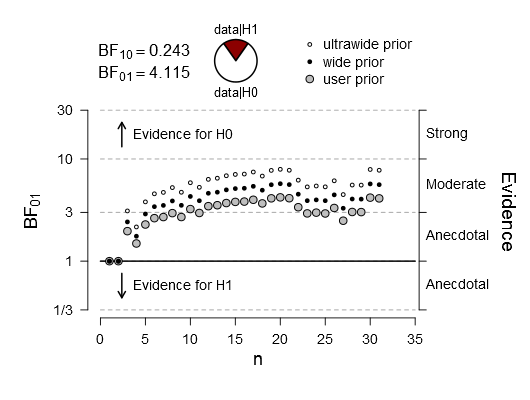


## Choose C

##### Prior and Posterior


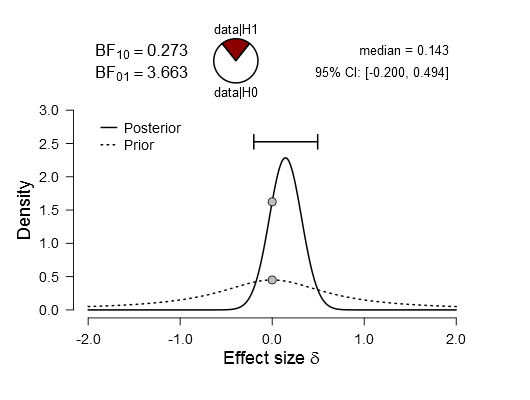


##### Bayes Factor Robustness Check


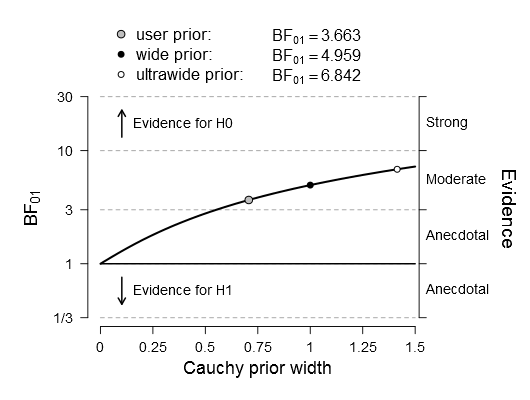


##### Sequential Analysis


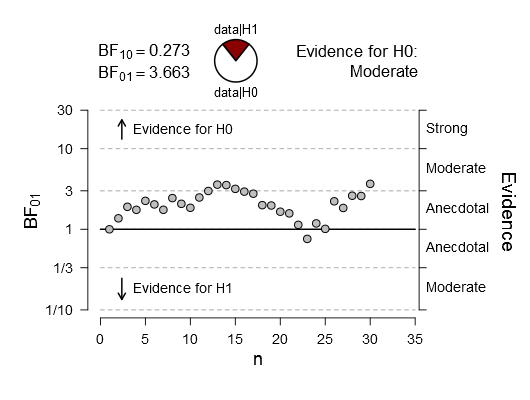


## Choose D

##### Prior and Posterior


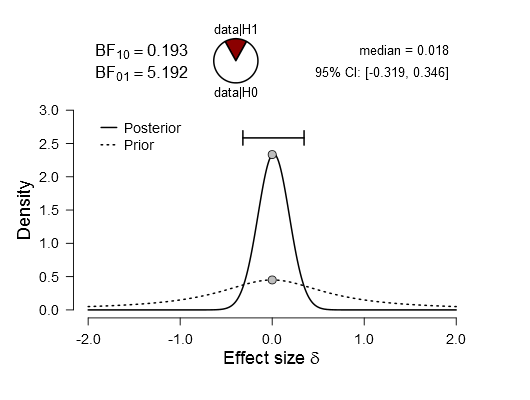


##### Bayes Factor Robustness Check


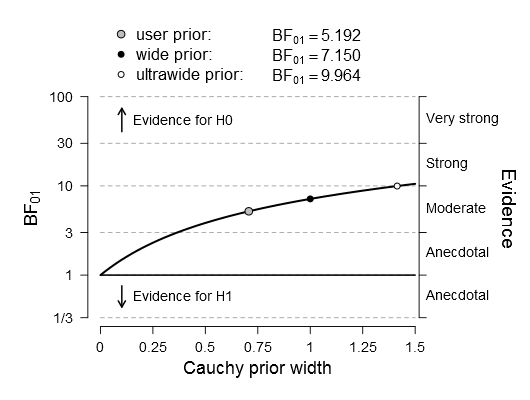


##### Sequential Analysis


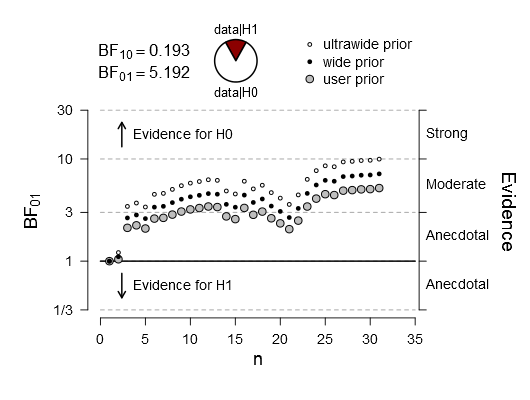


## Choose E

##### Prior and Posterior


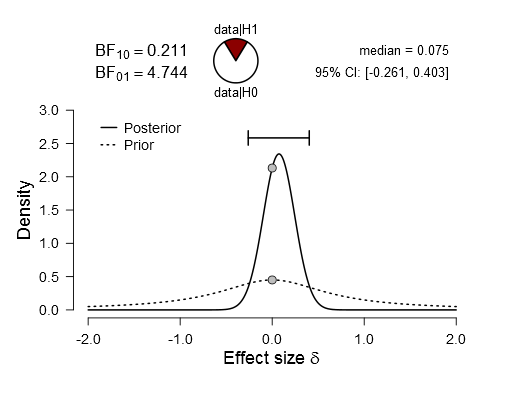


##### Bayes Factor Robustness Check


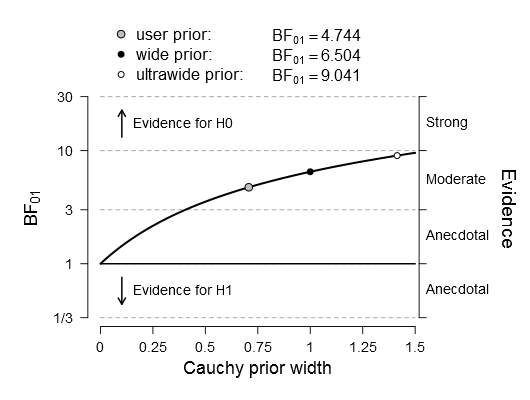


##### Sequential Analysis


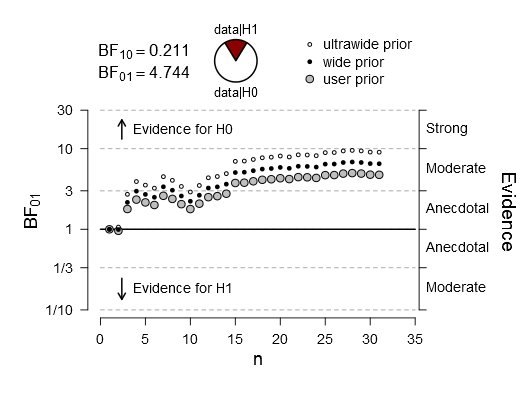


## Choose F

##### Prior and Posterior


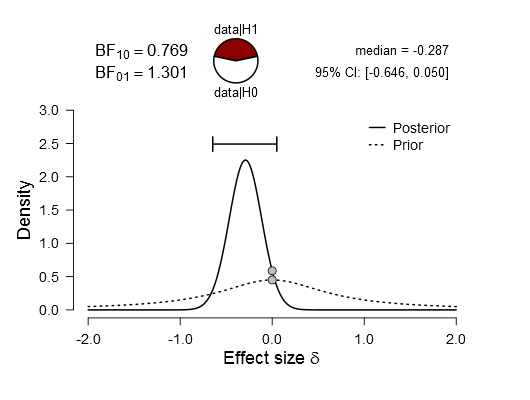


##### Bayes Factor Robustness Check


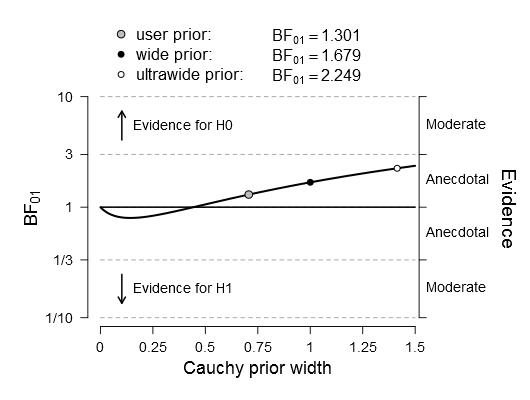


##### Sequential Analysis


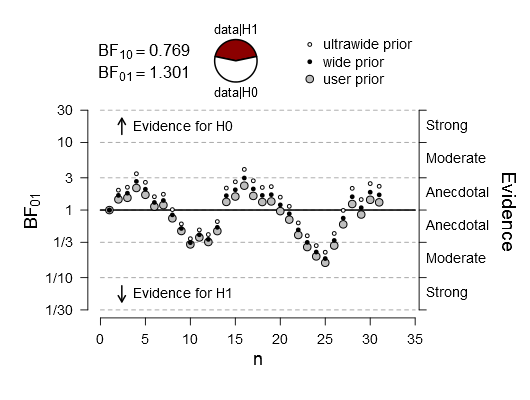


# Inclusion/Exclusion Criteria

The drugs used in the study had strict contraindications which formed part of the exclusion criteria. The full exclusion/inclusion criteria are presented here:

Participants must:

- be 65 years or older
- have good eyesight (corrected or otherwise)
- be a native English speaker

Participants must not have:

- any neurological or psychiatric problems
- a known sensitivity to levodopa, benserazide, domperidone or vitamin C (the placebo tablet used)
- lactose intolerance, galactosemia or glucose/galactose malabsorption
- Huntington’s Chorea
- intention tremor
- prolactin-releasing pituitary tumour (prolactinoma)
- glaucoma
- a history of malignant melanoma
- a suspicious, undiagnosed lesion
- peptic ulcers
- unstable diabetes
- osteomalacia
- severe endocrine, hepatic, renal, pulmonary or cardiac disorders
- electrolyte disturbances
- prolongation of cardiac conduction intervals

Participants must not be taking:

- dopaminergic, noradrenergic, serotonergic or anticholinergic medications
- monoamine oxidase inhibitors (MAO-I) or cholinesterase inhibitors
- antihypertensive (blood pressure) drugs containing reserpine
- ferrous sulphate
- antacids
- opioids or sympathomimetics (e.g. amphetamines, epinephrine/adrenaline)
- diazepam
- neuroleptics
- ketoconazole, erythromycin or any CYP3A4 inhibitors e.g. fluconazole, voriconazole, clarithromycin, amiodarone, telithryomycin
- antibiotics – e.g. erythromycin, levofloxacin, moxifloxacin, spiramycin
- anti-fungal agents – pentamidine
- anti-malarial agents – esp. halofantrine, lumefantrine
- gastro-intestinal medicines – cisapride, dolasetron, prucalopride
- antihistaminics – mequitazine, mizolastine
- treatments used in cancer – e.g. toremifene, vandetanib, nivamine
- AIDS/HIV medications – protease inhibitors

# Task instructions

The following instructions were printed on the screen before the start of the learning and practice trials:

1. ‘In each trial you have to choose between the two symbols displayed on the screen, to either side of the central cross. Your choice will be circled in red. To choose the left symbol, press button 1. To choose the right symbol, press button 5. Please press key 3 to continue'
2. 'As an outcome of your choice you may
   - 1. -get nothing
     2. -gain a coin
     3. -lose a coin
   1. Please press key 3 to continue'
3. 'The two symbols displayed on the same screen are not equivalent in terms of outcome: with one you are more likely to get nothing than the other. Each symbol has its own meaning, regardless of where and when it is displayed. The aim of the game is to win as much money as possible. Please press key 3 to continue’
4. This is the beginning of the first block. Use keys 1 and 5 to respond Please press key 3 to begin'

The experimenter then provided the following instructions verbally:

‘You’ll see 2 symbols on the screen, and you’ll select one. Then you’ll have one of 4 outcomes – you’ll either:

- win 20p
- lose 20p
- just see a picture of 20p but not win or lose
- or see nothing, win and lose nothing

I can’t tell you how the task works, because it’s an implicit/subconscious learning task, so hopefully as the task goes along you’ll start to get better and maybe pick up on the rules. I can say that it’s not a repeating sequence, so it doesn’t go ‘this symbol twice and then that symbol 3 times’, and that it does depend on the specific symbols on the screen so you have to pay attention to them. If you’re unsure you should trust any gut feelings you have towards one of the symbols.’

The following instructions were shown on the screen before the novel pairs test began:

1. 'In each trial you have to choose between the two symbols displayed on the screen, to either side of the central cross. Your choice will be circled in red.
   - to choose left, press 1
   - to choose right, press 5

Please press key 3 to continue'

1. 'You get the same outcomes as on the previous test:

- get nothing
- gain a coin
- lose a coin

but this time there will not be shown feedback (i.e. you will still be winning and losing money, we just won't be telling you when you do). Please press key 3 to continue'

1. 'The two symbols displayed on the same screen are not equivalent in terms of outcome: with one you are more likely to get nothing than the other. Each symbol has its own meaning, regardless of where and when it is displayed. The aim of the game is to win as much money as possible. Please press key 3 to continue'

The experimenter then provided the following verbal instructions:

‘In this block you won’t be shown the feedback after you select the symbol, so you won’t find out if you’re correct or not, but you will still be winning and losing money. This is a test to see how well you learnt the task, so you should use what you learned on the learning blocks.

Also, the symbols will be shown in new combinations, so you can have a symbol from one pair shown with a symbol from the other pair.’
